# Supplementary figures and images for: Comparing the epigenetic landscape in myonuclei purified with a PCM1 antibody from a fast/glycolytic and a slow/oxidative muscle
Source: PLoS Genet. 2021 Nov 9;17(11):e1009907. doi: 10.1371/journal.pgen.1009907 (PMC8604348; doi:10.1371/journal.pgen.1009907)

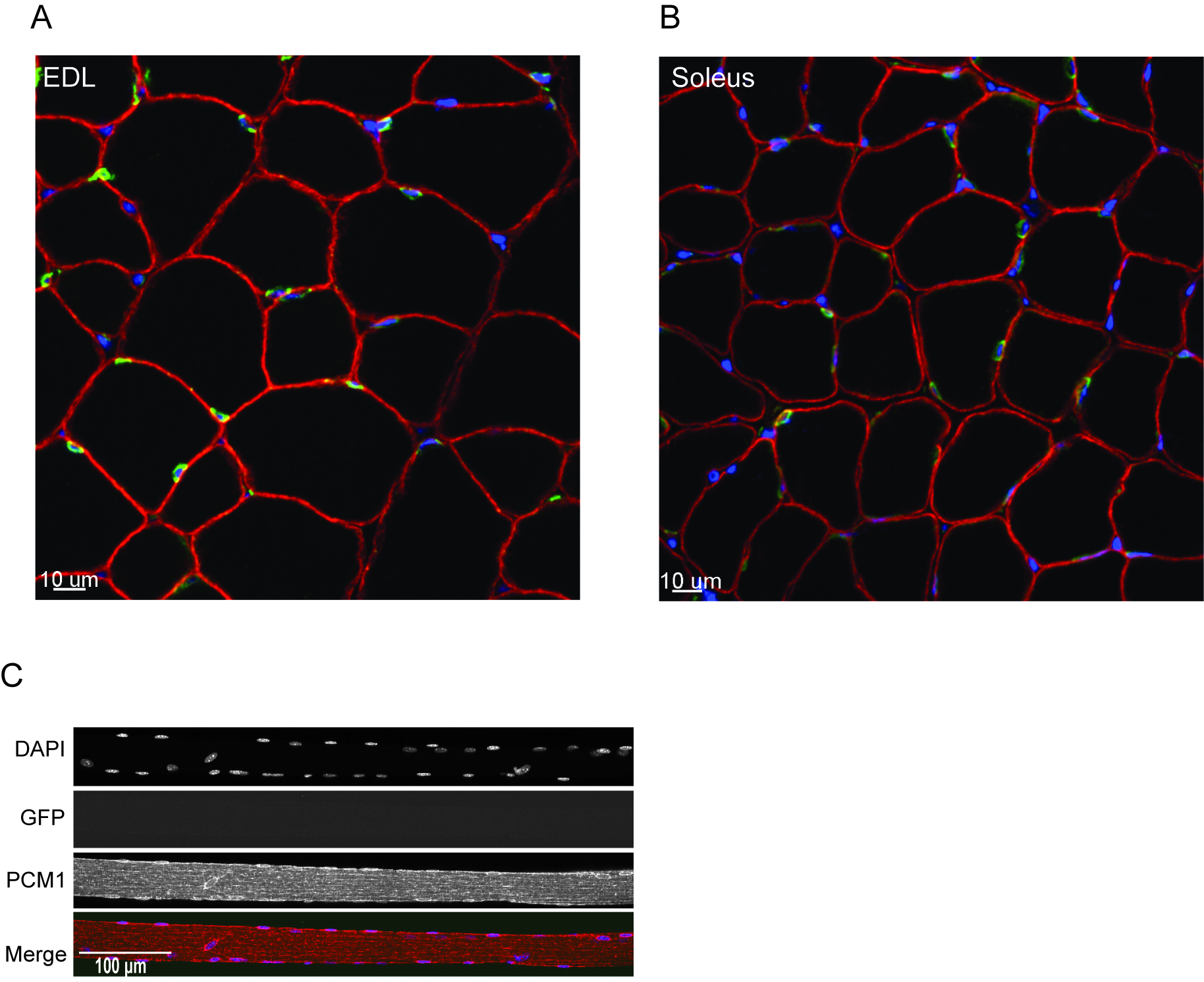

Supplement: S1 Fig — (A-B) Cross-section of EDL and soleus muscles stained with antibody against PCM1 (green), dystrophin (red) marking the boundary of the myofibers. Counterstained with DAPI to visualize DNA (Blue). Scale bar 10 μm. (C) Max intensity projection of a single fiber from a wild-type control mouse corresponding to the GFP and PCM1 co-localization in Fig 1. Scale bar 100 μm. (TIF) [file pgen.1009907.s001.tif]

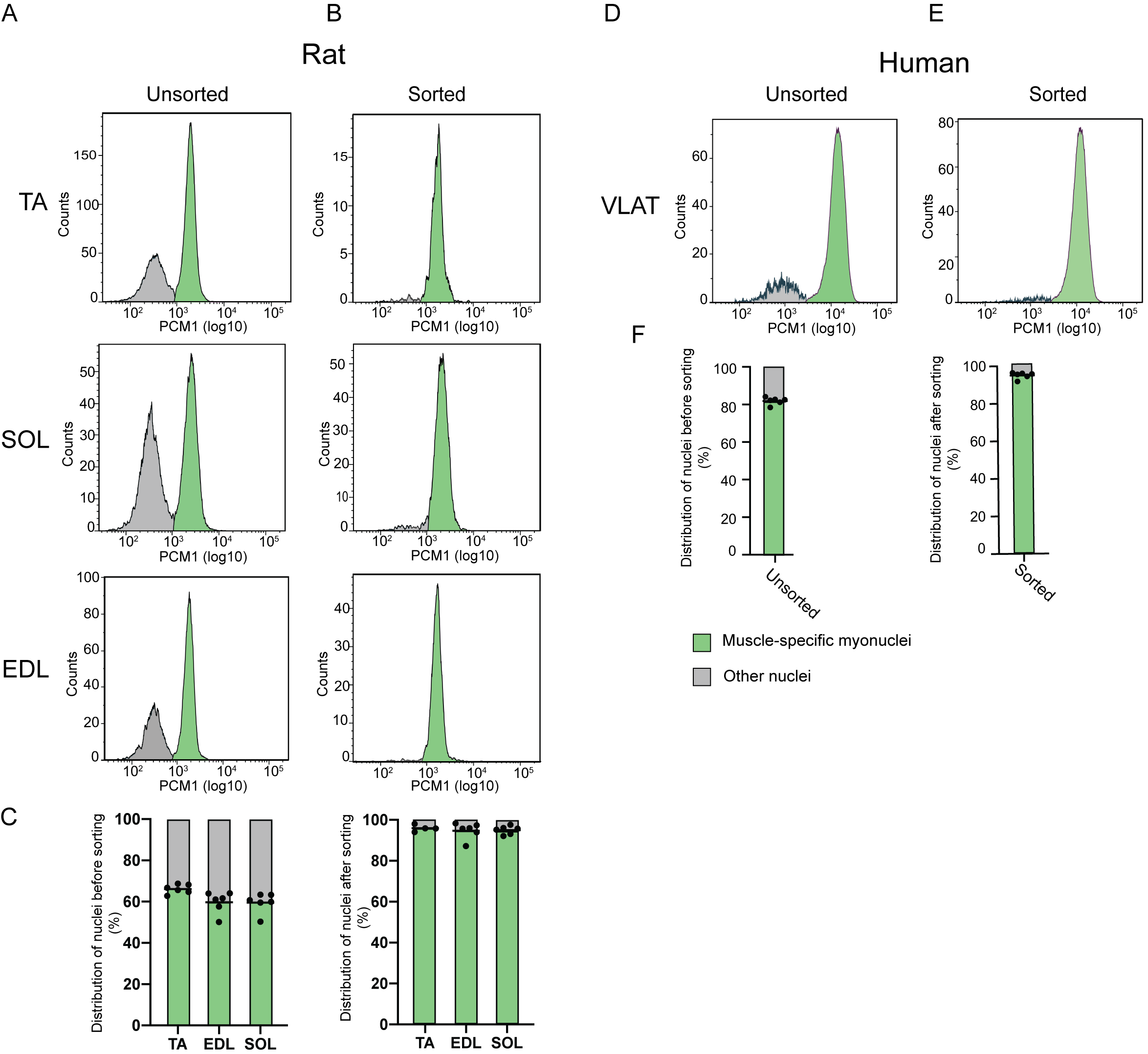

Supplement: S2 Fig — (A-B) Representative histograms of nuclei distribution and magnetic sorting efficiency for the three muscles TA, EDL and soleus (SOL) in rats analyzed by flow cytometry. (C) Quantification of nuclei distribution and sorting efficiency in rats. (D-E) Representative histograms of nuclei distribution and magnetic sorting efficiency in human biopsies from vastus lateralis (VLAT) analyzed by flow cytometry. (F) Quantification of nuclei distribution and sorting efficiency in humans (n = 4–6). (TIF) [file pgen.1009907.s002.tif]

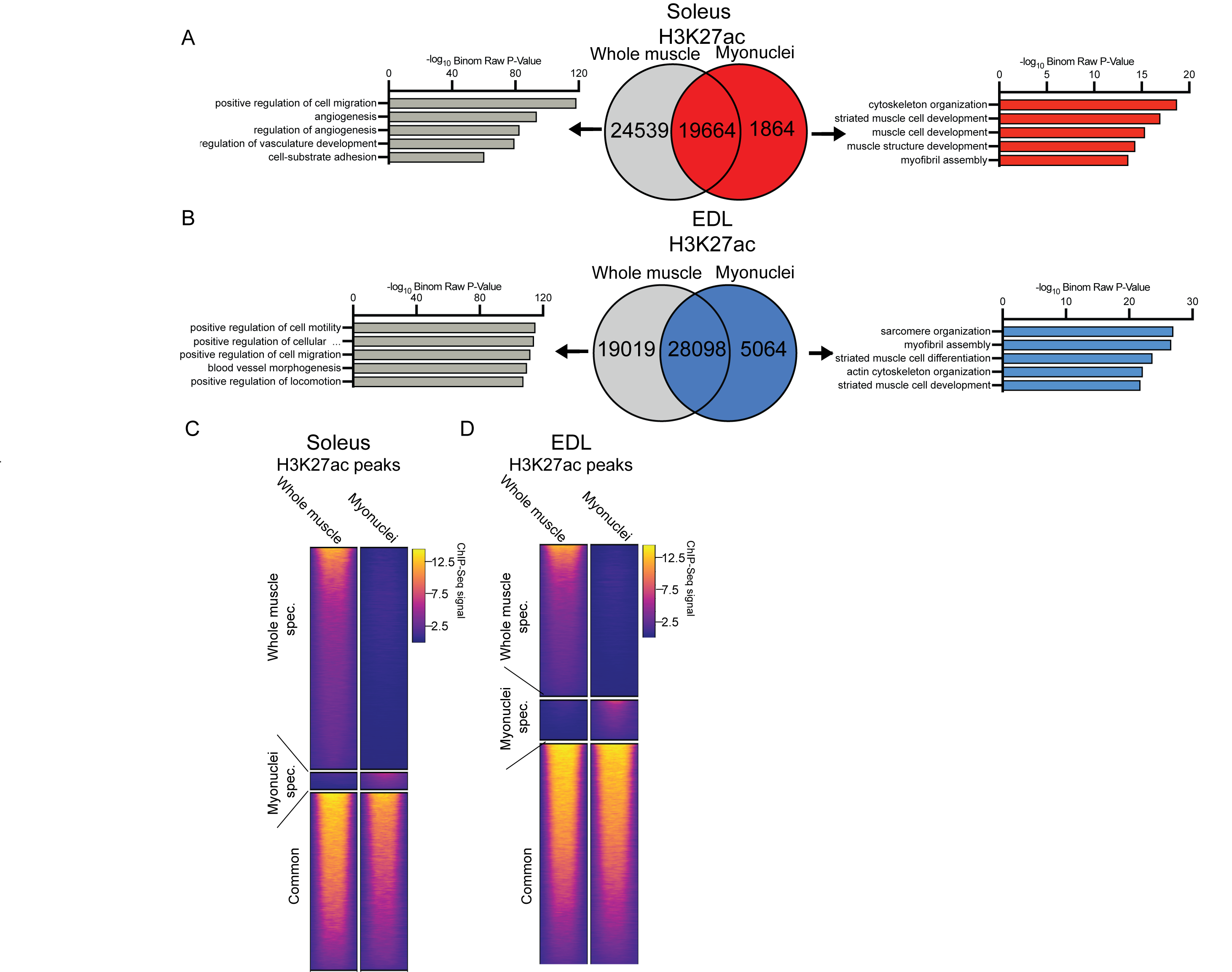

Supplement: S3 Fig — (A-B) Venn diagram of unique and common H3K27ac peaks between soleus and EDL whole muscle and myonuclei, respectively. The five most enriched gene ontologies for the unique peaks are shown to the left (whole tissue) and right (myonuclei). For full list of ontologies see S2A–S2D Table. Whole muscle H3K27ac data from [42]. Gene ontology identified with GREAT [143] using single closest gene. (C-D) Heatmaps of enrichment in H3K27ac peaks in whole muscle and myonuclei in soleus and EDL, respectively. (TIF) [file pgen.1009907.s003.tif]

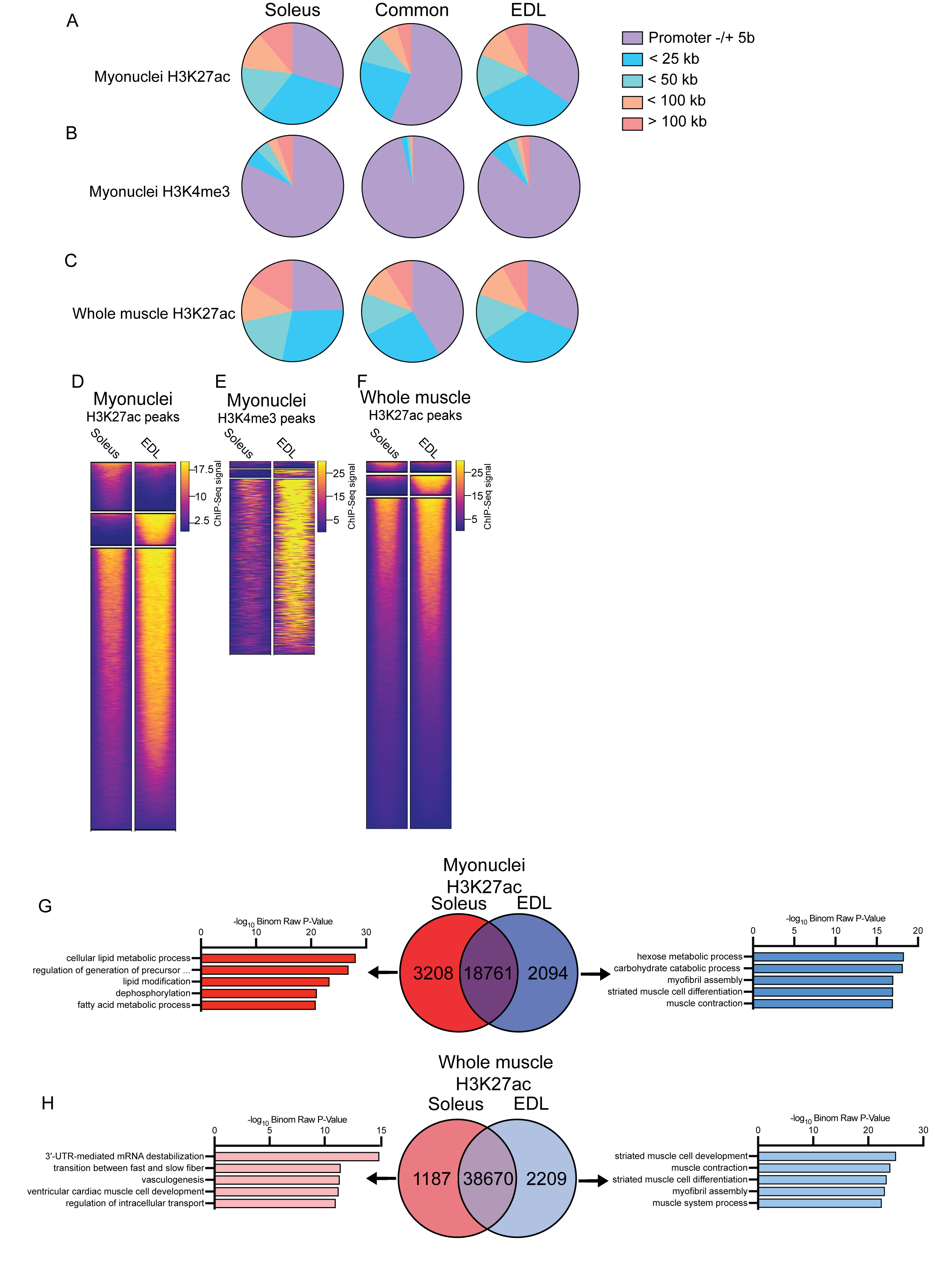

Supplement: S4 Fig — (A-C) Genomic distribution of differently enriched peaks between soleus and EDL. (D-F) Heat map of the differently enriched and common peaks for H3K27ac and H3K4me3 in myonuclei and H3K27ac in whole muscle. (G-H) Venn diagram showing overlap between H3K27ac for soleus and EDL in myonuclei and whole muscle, respectively. Five most enriched gene ontologies for specific H3K27ac peaks are shown. For full list of ontologies see S3A–S3D Table. Whole muscle H3K27ac data obtained from [42]. Gene ontology identified with GREAT [143] using single closest gene. (TIF) [file pgen.1009907.s004.tif]

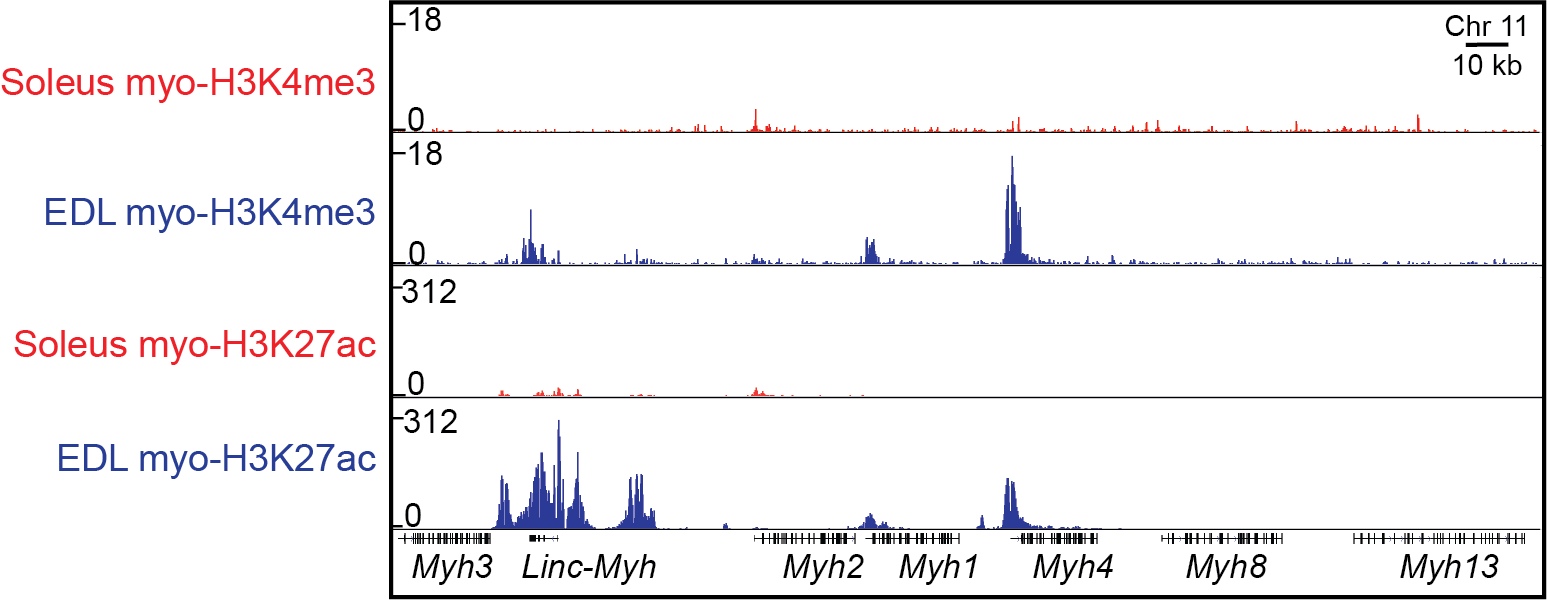

Supplement: S5 Fig — ChIP-Seq profiles of the myo-specific H3K4me3 and H3K27ac enrichment at the MyHC locus at chromosome 11 encoding the embryonic myosin Myh3 (MyHC-emb), the noncoding RNA Linc-Myh, the adult versions Myh2 (MyHC-2A), Myh1 (MyHC-2X), Myh4 (MyHC-2B), neonatal myosin Myh8 (MyHC-neo) and the extraocular myosin Myh13 (MyHC-EO). (TIF) [file pgen.1009907.s005.tif]

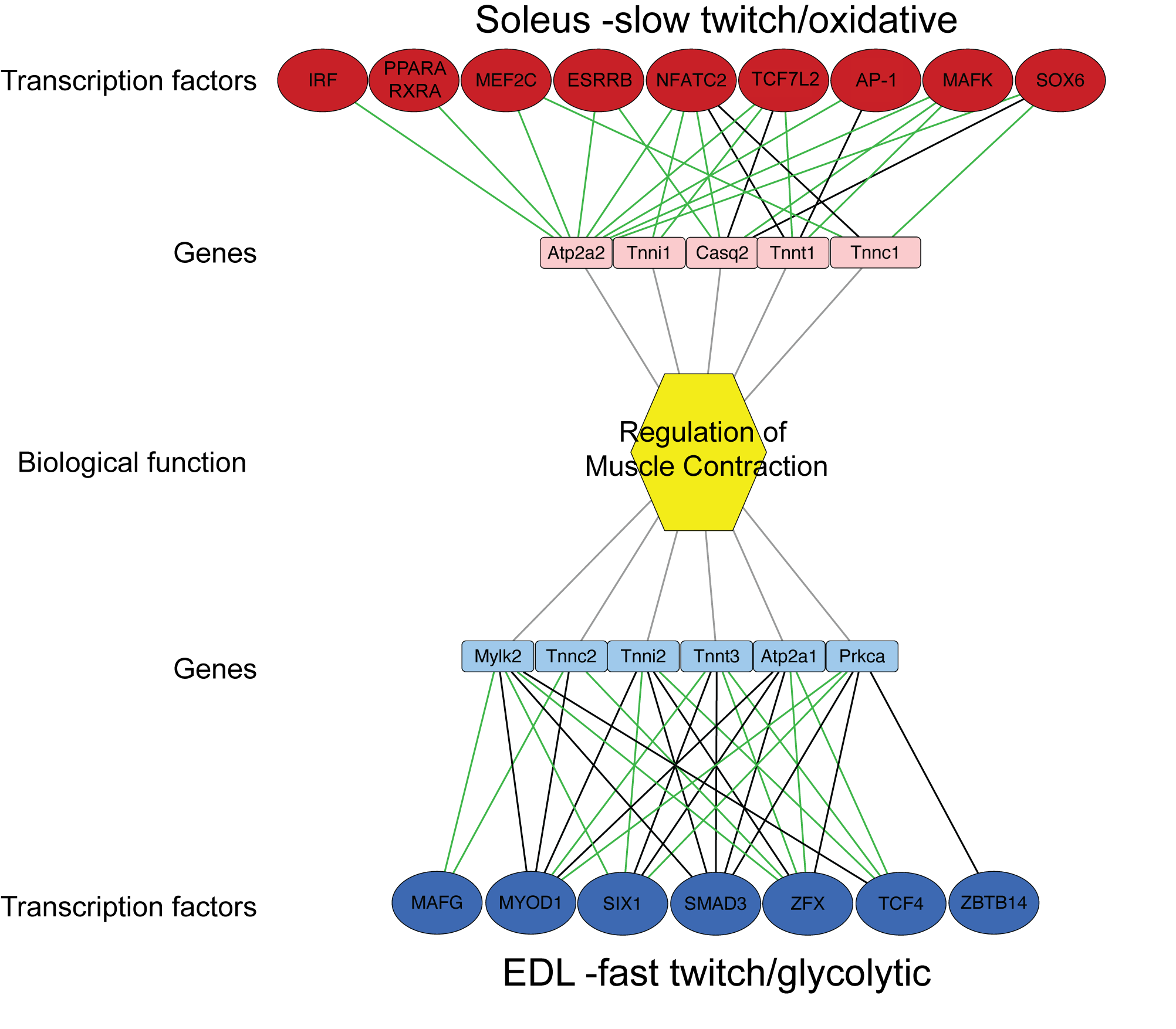

Supplement: S6 Fig — Transcriptional regulatory network in muscle contraction for soleus (Top) and EDL (bottom). Closest genes with DE promoter for genes involved in regulation of muscle contraction inside 100 kb of the predicted binding motif. Color of the edges, black and green, indicates position of most significant motif prediction for factor at promotor region or distal regulatory enhancer region, respectively. (TIF) [file pgen.1009907.s006.tif]
